# Autoantibodies and Malaria: Where We Stand? Insights Into Pathogenesis and Protection

**DOI:** 10.3389/fcimb.2020.00262

**Published:** 2020-06-11

**Authors:** Luiza Carvalho Mourão, Gustavo Pereira Cardoso-Oliveira, Érika Martins Braga

**Affiliations:** Departamento de Parasitologia, Instituto de Ciências Biológicas, Universidade Federal de Minas Gerais, Belo Horizonte, Brazil

**Keywords:** malaria, autoantibodies, anemia, cerebral malaria, renal dysfunction

## Abstract

Autoantibodies are frequently reported in patients with malaria, but whether they contribute to protection or to pathology is an issue of debate. A large body of evidence indicates that antibodies against host-self components are associated to malaria clinical outcomes such as cerebral malaria, renal dysfunction and anemia. Nonetheless, self-reactive immunoglobulins induced during an infection can also mediate protection. In light of these controversies, we summarize here the latest findings in our understanding of autoimmune responses in malaria, focusing on *Plasmodium falciparum* and *Plasmodium vivax*. We review the main targets of self-antibody responses in malaria as well as the current, but still limited, knowledge of their role in disease pathogenesis or protection.

## Introduction

Despite substantial progress in control efforts over the past decades, malaria still accounts for significant morbidity and mortality, mainly in underdeveloped countries. In 2018, an estimated 228 million cases of malaria occurred worldwide with 405,000 deaths, largely in Africa (WHO | World Malaria Report, 2019). Five species are known to cause malaria in humans, *Plasmodium falciparum, Plasmodium vivax, Plasmodium knowlesi, Plasmodium ovale*, and *Plasmodium malariae*. Since research emphasis has been placed on *P. falciparum* and *P. vivax*, parasites that are responsible for most of malaria cases, here we will focus in these two species.

Symptomatic disease occurs during the erythrocytic phase when the presence of asexual blood-stage parasites triggers a robust innate immune response. This response if properly regulated may clear infection, contributing to the development of a protective immunity. By the other hand, if not counterbalanced by anti-inflammatory responses, the exacerbated activation of the immune system may play a key role in the pathogenesis (reviewed by Antonelli et al., 2019), leading to complications such as cerebral malaria, anemia, acute kidney injury and respiratory distress syndrome (Moxon et al., 2019).

During infection, high levels of antibodies with a broad range of specificities are elicited. Although their functional activity is far from over, it is known that such molecules can have diverse effects. Antibodies are critical for the control of the disease by acting alone or in cooperation with host immune cells (For further details see Teo et al., 2016). But in some cases, antibodies that recognize host's own components may also promote pathology (Ludwig et al., 2017).

The presence of autoantibodies that recognize the host's own molecules has also been extensively reported in patients with malaria (Rosenberg et al., 1973; Berzins et al., 1983; Daniel-Ribeiro et al., 1983; Wozencraft et al., 1990; Jakobsen et al., 1993; Lacerda et al., 2011; Fernandez-Arias et al., 2016; Mourão et al., 2016, 2018; Rivera-Correa et al., 2019a). The mechanisms by which autoimmune responses could be triggered during an infection remains unclear but it is generally accepted that they may include: molecular mimicry (Damian, 1964; Greenwood, 1974), bystander activation (Fujinami et al., 2006; Münz et al., 2009), epitope spreading (Vanderlugt and Miller, 2002; Münz et al., 2009), persistent infection and B cells polyclonal activation (Freeman and Parish, 1978; Rosenberg, 1978; Daniel-Ribeiro et al., 1983; Minoprio, 2001).

Molecular mimicry is the sharing of structurally similar antigens between parasite and host components (Damian, 1964). In malaria, molecular mimicry occurs between *P. falciparum* translationally controlled tumor protein (PfTCTP) and human histamine-releasing factor (HRF) (MaCDonald et al., 2001). Another plasmodial protein that share motifs with host's components is *P. falciparum* erythrocyte membrane protein 1 (PfEMP1), which exhibits homology with human vitronectin (Ludin et al., 2011). An *in silico* analysis comparing *P. vivax* entire proteome and human RBC proteome also revealed that 23 *P. vivax* proteins shared similarity to human RBC proteins such as ankyrin, actin, and spectrin (Mourão et al., 2018). These structural similarities can activate cross-reactive autoreactive lymphocytes, consequently disordering the immune system. So, when T- or B- cells receptors recognize a parasite epitope that is similar enough to a self-protein, an autoimmune response is elicited, leading to cell or tissue destruction in addition to activation of other branches of the immune system (Fujinami et al., 2006; Münz et al., 2009).

Bystander activation is an antigen-independent phenomenon whereby parasitized cells, either through direct cell contact or paracrine signals, alert or instruct neighboring non-infected cells to produce inflammatory mediators (Holmgren et al., 2017). The inflammatory milieu evoked by the infection promotes the activation and expansion of autoreactive T or B cells, which can initiate an autoimmune response that damage host's cells or tissues, leading to the release of self-reactive antigens (Fujinami et al., 2006; Münz et al., 2009). Evidences of bystander activation in malaria came from *in vitro* studies investigating the pathways driving inflammation in infection. These studies have demonstrated that extracellular vesicles derived from plasma of mice infected with *Plasmodium berghei* or from *P*. *falciparum*-infected erythrocytes were able to activate naïve host cells (Couper et al., 2010; Mantel et al., 2013).

It is widely known that in early immune responses, epitopes of the initial antigens are recognized by the acquired immune system, but during infection, epitopes other than the dominant ones may also become immunogenic and be targets of T and B cells. This reactivity to newer endogenous epitopes is termed “epitope spreading” and may be induced against other epitopes in the same autoantigen (intramolecular epitope spreading) or against epitopes in other self-antigens (intermolecular epitope spreading) that are released after T- or B-cell-mediated bystander (Münz et al., 2009). Although epitope spreading is more commonly reported in autoimmune diseases, it may also occur in persistent infections, as it has been suggested by Flanagan et al. (2006) in a study conducted with adults naturally exposed to malaria in Kenya. These authors have investigated cellular immunity to the thrombospondin-related adhesive protein of *P. falciparum* (PfTRAP) and showed that the immunodominant response stimulated in the primary exposure to this protein has progressed to encompass lesser epitopes with repeated and prolonged exposure.

*In vitro* experiments with *P. falciparum*-infected RBCs revealed that culture supernatant containing parasite-derived products was able to induce polyclonal B-cell activation and non-specific immunoglobulin synthesis, suggesting that B-cell's proliferation and differentiation into antibody-secreting cells triggered by pathogen's molecules can also lead to autoimmune responses (Freeman and Parish, 1978; Minoprio, 2001). One of the molecules that has been incriminated as a potential activator of B-cells in malaria is the cysteine-rich interdomain region 1 (CIDR1) of *P*. *falciparum* erythrocyte membrane protein 1 (PfEMP-1). Evidence in this line has been provided by a study with B cells from non-immune donors stimulated with a recombinant version of CIDR1. The recombinant protein was able to promote *in vitro* proliferation, increase in B-cell size, and expression of immunoglobulins and cytokines in those cells (Donati et al., 2004). However, just a small proportion of antibodies secreted by them was specific for parasite antigens; the greater part was non-specific and could react with different host's components, leading to cell and tissue damage.

**Figure 1:**
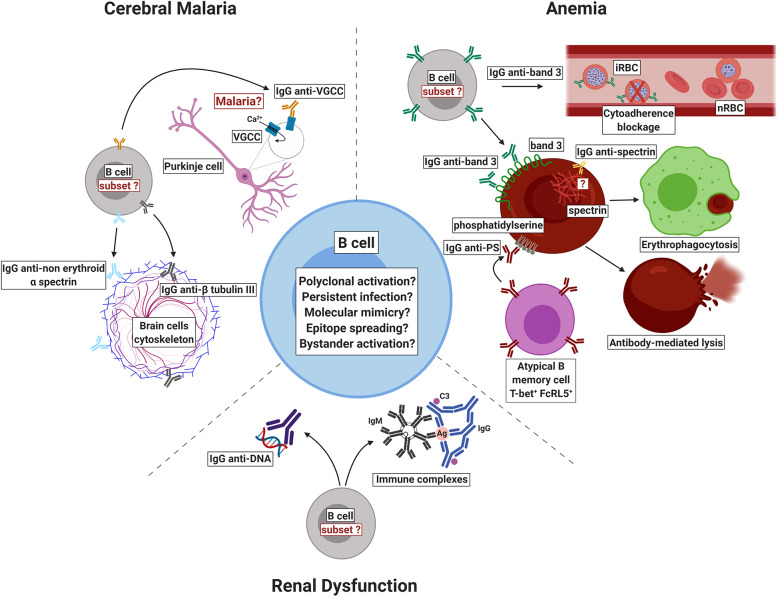
Schematic representation of self-reactive antibodies and their proposed role in the pathogenesis or protection against malaria. Autoantibodies that recognize host's own molecules have been reported in patients with malaria but the mechanisms by which such autoimmune response is induced are still to be completely elucidated. In infections due to *P. falciparum*, self-reactive immunoglobulins against voltage-gated calcium channel (VGCC), non-erythroid alpha spectrin, and beta tubulin III have been associated to cerebral malaria. In *P. falciparum* and *P. vivax* infections, anti-phosphatidylserine antibodies (IgG anti-PS) have been shown to recognize non-infected red blood cells (nRBCs) exposing phosphatidylserine, mediating their clearance and contributing to malaria-associated anemia. During *P. vivax* malaria, autoimmune responses to anion exchanger 1 (band 3 protein) have been implicated in the removal of nRBCs by decreasing their deformability and enhancing their uptake by THP-1 cells. On the other hand, in *P. falciparum* malaria, anti-band 3 self-reactive antibodies have been associated to protection through the blockage of cytoadherence. Antibodies to erythrocytic spectrin are elicited during *P. vivax* malaria but it remains unclear how they bind to cytoplasmic proteins. It has been hypothesized that the inflammation triggered by infection may damage brain cells, leading to the exposure of spectrin, which may activate the complement, amplifying neuronal damage. Finally, anti-DNA autoantibodies and immune complexes containing self-immunoglobulins have been suggested to play a role in renal dysfunction by depositing in renal tissues. iRBC: infected red blood cell. Ag: antigen. C3: complement component 3. Figure created with BioRender.

Self-reactive antibodies recognize different self-antigens such as erythrocyte proteins (Rosenberg et al., 1973; Fontaine et al., 2010; Mourão et al., 2016, 2018; Ventura et al., 2018), brain molecules (Bansal et al., 2009; Gitau et al., 2013), phospholipids (Adebajo et al., 1993; Jakobsen et al., 1993; Facer and Agiostratidou, 1994; Fernandez-Arias et al., 2016; Barber et al., 2019; Rivera-Correa et al., 2019a,b), and nucleic acids (Adu et al., 1982; Adebajo et al., 1993; Rivera-Correa et al., 2019b). Although the literature reporting the detection of autoantibodies in plasmodial infections is vast, the role of such molecules in malaria is still a controversial issue. Some authors have associated such autoimmune responses to pathology while others to protection. In this review, we summarize the latest breakthroughs regarding autoantibody responses in malaria, emphasizing what is new on the pathogenesis front, mainly with respect to cerebral malaria, kidney injury and anemia (Figure 1).

## Cerebral Malaria

Cerebral malaria (CM) is a clinical syndrome of severe falciparum malaria characterized by impaired consciousness assessed by Blantyre Coma Score ≤ 2 in children (Molyneux et al., 1989) or Glasgow Coma Score ≤ 11 in adults (Teasdale and Jennett, 1974), with no other cause of encephalitis (Taylor et al., 2004). Although this neurological syndrome only develops in a small percentage of *P. falciparum*-infected patients, it is responsible for more than 90% of malaria-related deaths. The treatment with anti-malarial drugs decreases mortality due to CM, but nearly 20% of treated patients still succumb and up to one-third of survivors frequently exhibit long-term neurological sequelae such as cognition and speech disorders, physical disability and cortical blindness (Birbeck et al., 2010).

Although the mechanisms leading to CM pathogenesis are not yet clearly defined, it is known that both parasite and host factors play a role in the clinical outcome of this syndrome (Idro et al., 2010). Among host components, B cells and antibodies are critical for the immune response against malaria. Large amounts of antibodies are produced in response to plasmodial infection, including those that recognize self-components such as host brain antigens (Guiyedi et al., 2007; Bansal et al., 2009; Duarte et al., 2012). However, whether such self-reactive immunoglobulins are a consequence of cerebral malaria or a factor that aggravates the disease is little explored. This could be, in part, due to the difficult in accessing human brain tissues because of the small proportion of *P. falciparum*-infected patients that develop CM. Moreover, the existence of ethical issues limits the study of CM to peripheral blood and *post-mortem* samples.

Although understudied, some research groups have associated a marked increase in specific anti-brain autoantibodies levels with disease severity in *P. falciparum* malaria. This is the case of self-reactive antibodies against the voltage-gated calcium channels (VGCCs), whose levels were shown to be higher in Kenyan children with CM than in those with uncomplicated disease or uninfected (Lang et al., 2005). Autoantibodies to VGCCs have been shown to downregulate calcium flow in Purkinje neurons and granule cells through a complement-independent process in autoimmune diseases such as limbic encephalitis and cerebellar ataxia (Pinto et al., 1998; Irani and Lang, 2008), thereby providing insight into the pathogenic role of such self-reactive molecules in malaria.

Furthermore, it has been reported that serum from Gabonese children with severe *P. falciparum* infection recognize a higher diversity of brain antigens in comparison to non-infected ones. Some of those autoantibodies display reactivity to the non-erythroid alpha spectrin (Guiyedi et al., 2007), a structural protein that is found in the cytoplasm of a variety of brain cells and is responsible for membrane structure and integrity. Since inflammation induced by malaria can damage brain cells exposing non-erythroid alpha spectrin, it is possible that this protein activates the complement system, amplifying the neuronal damage. In primary Sjögren's syndrome, an important autoimmune disease, non-erythroid spectrin undergoes proteolysis by caspase 3 and calpain, producing a fragment that acts as autoantigen (Nath et al., 1996; Haneji et al., 1997).

Other cytoskeletal protein that has also been described as a target of autoimmune responses in CM and which is considered as a disease-specific marker is beta tubulin III (TBB3) (Bansal et al., 2009), a protein that is abundant in cells from nervous system and in neoplastic cells of neural tumors (Katsetos et al., 2003). TBB3 is involved in axon guidance, thus mutations in this protein are associated with different nervous system disorders (Tischfield et al., 2010). In living cells and *in vitro* models, polyclonal antibodies with high affinity for beta tubulin have been shown to disrupt cytoplasmic microtubules, leading to their fragmentation into smaller units (Füchtbauer et al., 1985).

In addition to the self-reactive proteins mentioned above, the dendritic tree of Purkinje cell is another host component that has been considered a target of autoimmune responses in CM. In a cohort of Thai individuals, it has been demonstrated that levels of autoantibodies against dendrites are higher in *P. falciparum*-infected patients with CM than in those with uncomplicated malaria (Gallien et al., 2011). The pathogenic role of such autoantibodies was attributed to their ability in inhibiting *in vitro* development of Purkinje cells (Calvet et al., 1993). However, it is important to emphasize that these results were obtained from studies conducted with cat brain biopsies and thus, should be interpreted with caution.

On the other hand, a possible role in protection against severe *P. falciparum* malaria has already been suggested for self-reactive antibodies induced during plasmodial infection. This is the case of IgE autoantibodies to 14-3-3 ε brain protein, which induce *in vitro* mastocyte degranulation (Duarte et al., 2012). The 14-3-3 ε brain protein belongs to a family of adaptor proteins that interact with a multitude of binding partners that contain PSer/PThr motifs. Through this interaction, 14-3-3 ε protein affects the activity and localization of various substrate proteins, regulating signal cascades of a wide range of biological activities, including cell cycle and apoptosis (Cornell and Toyo-Oka, 2017). Therefore, 14-3-3 ε protein has been implicated in different neurodegenerative and neuropsychiatric diseases by mechanisms that vary from apoptosis to protein stabilization and aggregation (For further details see Foote and Zhou, 2012 and Cornell and Toyo-Oka, 2017). In Parkinson's disease, for example, the interaction of 14-3-3 ε protein with Bad and Bax proteins prevents neurons apoptosis. Neurodegeneration is avoided by interaction between 14-3-3 ε protein and phosphorylated tyrosine hydroxylase in parallel with the binding between α-synuclein to unphosphorylated tyrosine hydroxylase. An imbalance in those interactions leads to neurodegeneration in this nervous system disorder (Shimada et al., 2013). Thus, it is not surprising that an antibody response against 14-3-3 ε brain protein exert neuroprotective properties, as it has already been demonstrated in glaucoma, using a neuro-retinal cell line of mouse origin. In this case, cell viability and reduced reactive oxygen species levels were considered predictors of protection (Bell et al., 2015).

As highlighted herein, the repertoire of brain antigens that are targets of autoimmune responses during CM is vast and depends on a complex interplay of host, parasite and environmental factors. However, whether such autoantibodies have a pathogenic relevance, can be considered biomarkers of neuropathology or are merely innocent by-standers remains a focus of debate. More studies are needed in order to elucidate this, as well as to determine the epitopes, function and origin of such self-reactive immunoglobulins. Even though it is known that a breach in the blood brain barrier's (BBB) integrity is necessary to allow antibody influx into the brain (Huerta et al., 2006), studies conducted with *post mortem* brain tissues from Malawian children with fatal cerebral malaria revealed that, although BBB breakdown occurs in vessels containing cytoadherent parasitized RBCs, no gross leakage of plasma proteins occurs (Brown et al., 2001). Thus, the mechanisms by which immunoglobulins gain access to brain tissue are another issue that is still to be elucidated. Do they cross the BBB independently or do plasma cells secrete them? Further studies are necessary to understand in more details how BBB breakdown occurs in malaria. Understanding how this happens may provide new opportunities to find agents that are able to open the BBB, allowing the delivery of different molecules and shedding light on the effects of antibodies in the brain tissue. This knowledge may pave the way for the development of future interventions for malaria and other neurological diseases.

## Renal Dysfunction

Acute renal failure is most reported in *P. falciparum* infections (Frutakul et al., 1974; Burchard et al., 2003; von Seidlein et al., 2012; Conroy et al., 2016; Sypniewska et al., 2017; Rivera-Correa et al., 2019b), but this complication can occasionally occurs in infection due to *P. malariae* (Neri et al., 2008; Badiane et al., 2016). Renal failure is considered a clinical manifestation with high prognostic value to severe malaria (von Seidlein et al., 2012; Sypniewska et al., 2017). In *P. malariae* infection, renal failure affects most children and is presented as steroid-resistant nephrotic syndrome. The pathogenesis is possibly mediated through immune-complex deposition containing IgM, IgG, C3, and malarial antigens in mesangiocapillary, glomerular, proximal tubules and subendothelial kidney tissues, with rarely IgA deposition (Ward and Kibuka-Musoke, 1969; Houba et al., 1971; van Velthuysen and Florquin, 2000; Das, 2008). Chronic glomerular disease due to *P. malariae* infection is usually not reversible even after treatment, raising the hypothesis that genetic and environmental factors are also involved (Houba, 1979). Although it is well-known that *P. malariae*-associated renal impairment is caused mainly because of immune complex deposits, there are no studies investigating if autoantibodies are involved in this process.

In *P. falciparum* malaria, acute renal failure is a common and serious complication in non-immune adults and adolescents and is more frequent in patients from non-endemic regions (Barsoum, 2000; Elsheikha and Sheashaa, 2007; Nguansangiam et al., 2007), but it can also occur in pediatric severe malaria (Olowu and Adelusola, 2004; von Seidlein et al., 2012; Conroy et al., 2016; Sypniewska et al., 2017; Rivera-Correa et al., 2019b). Although it is an important clinical manifestation associated with mortality and morbidity, the pathogenesis of renal failure in *P. falciparum* malaria is not well understood. However, unlikely *P. malariae*-associated renal failure, acute kidney injury in *P. falciparum* infection is usually transient and disappears after treatment (van Velthuysen and Florquin, 2000), suggesting that the parasite does not have a great role in the pathogenesis that is most likely to be caused by host's immune response. Several hypothesis on the pathogenesis of malarial renal failure have been proposed, including mechanical obstruction of glomerular and tubulointerstitial capillaries by infected erythrocytes (Seydel et al., 2006; Nguansangiam et al., 2007), possibly leading to renal ischemia (Conroy et al., 2016); immune complex deposits leading to renal impairment (Frutakul et al., 1974); and autoantibodies against nucleic acids (Wozencraft et al., 1990; Rivera-Correa et al., 2019b).

In a children population from Uganda, Rivera-Correa et al. (2019b) demonstrated that infants with severe *P. falciparum* malaria manifesting acute kidney injury have autoantibodies against nucleic acid and lipids. Additionally, they found a correlation between those autoantibodies and creatinine and blood urea nitrogen levels, two indicators of kidney health, suggesting that such immunoglobulins may play a role in kidney injury. It was also shown that anti-DNA autoantibodies were elevated in children with acute kidney injury, a result that is in accordance with Wozencraft et al. (1990), who obtained similar data, however, in mouse malaria. It is important to mention that no difference was found in levels of antibodies against parasite antigen, indicating that systemic changes in IgG metabolism and immune-mediated pathways may contribute to malaria-associated renal failure. This result corroborates the findings from Frutakul et al. (1974), who reported an absence of antibodies against parasite antigens in immune complexes deposited in glomeruli capillary walls from a Thai child's kidney. All these data demonstrate that renal dysfunction due to autoantibodies may be relevant in severe *P. falciparum*-associated renal failure. Future investigations should be conducted to further understand the role of those autoantibodies, their involvement in renal pathogenesis, as well as their use as disease biomarkers.

## Anemia

Anemia is the most common feature and a major concern in malaria, mainly in young children and pregnant women (Accrombessi et al., 2015; Kenangalem et al., 2016; White, 2018). Despite its relevance, the pathogenesis of malaria-associated anemia is complex, and still incompletely understood. Malaria-induced anemia is thought to arise from the rupture of infected and non-infected red blood cells (nRBCs), as well as inappropriate erythropoiesis in the erythroid germinal centers (Douglas et al., 2012; White, 2018). But the greater loss is due to the clearance of nRBCs, which persist long after infection has resolved (Looareesuwan et al., 1987; Ritter et al., 1993; Collins et al., 2003; Douglas et al., 2012). An autoimmune component has been suggested to explain this removal, although the mechanisms underlying autoimmunity in malarial anemia have not been thoroughly explored (White, 2018; Rivera-Correa and Rodriguez, 2019). Self-reactive antibodies that recognize RBCs have been documented in plasmodial infections since 1970s, when host-serum components associated with the surface of nRBCs were detected in patients with malaria using different methodologies (Rosenberg et al., 1973; Facer et al., 1979; Berzins et al., 1983; Fernandez-Arias et al., 2016; Mourão et al., 2016). However, their roles in the pathophysiology of anemia have not been thoroughly explored. Evidence in this line is given by studies that have shown a reduction in RBC life span following the clearance of *P*. *falciparum* (Looareesuwan et al., 1987). This reduction in RBC survival time has been observed mainly in anemic patients and is associated with the deposition of complement containing immune complexes on RBCs surface (Rosenberg et al., 1973). Furthermore, it has been demonstrated that autoantibodies against triosephosphate isomerase purified from patients with *P. falciparum* malaria can bind to RBCs, promoting their lysis and activating complement cascade thereby, contributing to anemia (Ritter et al., 1993).

Since these early findings, autoantibodies with other specificities have already been identified and associated to anemia in malaria. This is the case of anti-phosphatidylserine (PS) antibodies, which were found to tag nRBCs exposing phosphatidylserine (Fernandez-Arias et al., 2016; Barber et al., 2019; Rivera-Correa et al., 2019a). These self-reactive immunoglobulins have been shown to increase *in vitro* phagocytosis and *in vivo* clearance of nRBCs, contributing to malarial anemia in a murine model (Fernandez-Arias et al., 2016). Moreover, a negative correlation between the magnitude of anti-PS antibodies and hemoglobin levels has been reported for patients infected with *P. falciparum* and *P. vivax* (Barber et al., 2019; Rivera-Correa et al., 2019a,b). In addition, it has been demonstrated that a population of atypical B cells, which is characterized by the expression of CD11c and T-bet, secretes anti-PS antibodies. The activation of these cells has been shown to be dependent of parasite DNA and different receptors have been suggested to be involved such as interferon-γ receptor (IFN-γR), B-cell receptor (BCR) and Toll-like receptor 9 (TLR9) (Rivera-Correa et al., 2017). However, the role of such atypical cells in human malaria was still unknown until a recent evidence has emerged from a study conducted with *P. falciparum*-infected returned travelers (Rivera-Correa et al., 2019a). In this study, it has been shown that FcRL5^+^T-bet^+^ B-cells are expanded in acute malaria. Additionally, it has been observed that naïve human peripheral blood mononuclear cells are able to produce anti-PS antibodies when stimulated with lysates of *P. falciparum*-infected RBCs, highlighting such atypical subset of memory B cells as a major promoter of autoimmune anemia in malaria. Besides anti-PS antibodies, self-reactive immunoglobulins triggered by other host cell targets are also involved in RBCs lysis, as it has been evidenced in a complement lysis assay using annexin V to block the binding of anti-PS antibodies to phosphatidylserine. After the binding of annexin to PS, RBC lysis could be partially inhibited by plasma from *P. falciparum*-infected patients (Rivera-Correa et al., 2019a).

Autoantibodies against RBCs have also been described for *P. vivax* infections (Mourão et al., 2016, 2018; Ventura et al., 2018; Barber et al., 2019). However, since this parasite has unique biological features that restricts its invasion to reticulocytes, lower densities of peripheral parasitemia are generally expected for infections due to *P. vivax* in comparison to *P. falciparum*. But despite this, *P. vivax* causes a greater loss of nRBCs. Thus, it is possible that the mechanisms leading to nRBCs removal in *P. vivax* malaria are distinct from those observed from *P. falciparum*. More work is needed to elucidate this.

Different erythrocytic antigens have been shown to be recognized by self-reactive immunoglobullins from anemic *P. vivax*-infected patients such as band 3 (Mourão et al., 2018), an anion exchanger protein which mediates the change of intracellular bicarbonate (${\text{HCO}}_{3}^{-}$) to extracellular chloride (Cl−) (Cordat and Reithmeier, 2014). Since IgGs purified from the same patients can bind to the surface of non-parasitized RBCs, increasing their rigidity and enhancing their clearance by THP-1 phagocytes (Mourão et al., 2018), it is also possible that anti-RBCs antibodies mediate malarial anemia through erythrophagocytosis or through decreasing RBC deformability (Mourão et al., 2016). Other possibility is the withdrawn from circulation by mechanisms like those tagging senescent RBCs for clearance (Lutz and Bogdanova, 2013), a hypothesis that should be better investigated.

Other RBC protein that has also been considered a target for autoimmune responses during *P. vivax* malaria is spectrin, although it is still unclear how anti-spectrin antibodies bind to an inner component of RBC membrane. Since *in silico* analysis revealed that human spectrin primary structure shares homology with a *P. vivax* hypothetical protein, it is possible that molecular mimicry drives autoimmune response against human spectrin (Mourão et al., 2018), a hypothesis that needs to be experimentally validated.

On the other hand, no association between anti-RBCs antibodies and anemia has been observed in a study conducted with *P. vivax*-infected children and adolescents from Pará, a State located in Brazilian Amazon (Ventura et al., 2018). A similar result was also found by (Fernandes et al., 2008), who evaluated the frequency of malarial anemia, as well as cytokines and autoantibodies levels, in an area in which *P. vivax* and *P. falciparum* coexists. It is important to mention that despite no significant association has been found in both studies, higher frequency of anti-RBCs antibodies has been reported in patients with malaria (Fernandes et al., 2008; Ventura et al., 2018).

In other reports, a beneficial role has been attributed to anti-RBCs antibodies. This is the case of a study carried out in an area of intense transmission of malaria in Liberia, where it has been shown that immune responses to band 3 neoantigens are correlated with lower *P. falciparum* parasitemia and can block *in vitro* and *in vivo* RBCs' cytoadherence (Hogh et al., 1994). Moreover, an anti-plasmodial activity has been proposed to autoantibodies from patients with autoimmune diseases, which were able to inhibit parasite growth, suggesting a protective role for those molecules, although the authors have not ruled out the involvement of other serum components (Brahimi et al., 2011). Since the pathways involved in autoantibody-induced pathology differ among infections due to different parasites, it is possible that self-reactive antibodies exert diverse effects in infections by *P. vivax* and *P. falciparum*, an issue that should be target of future investigation.

As can be noted by the findings mentioned above, the literature concerning self-reactive antibodies against RBCs suggest a dual role for these immunoglobulins in malaria-associated anemia. But crucial gaps remain to be addressed (Box 1).

Box 1 **Outstanding questions in autoimmunity-mediated pathology in malaria:**

- Which mechanisms are behind the generation of self-reactive antibodies in malaria?
- What are the self-antigens that trigger auto-immune responses in *P. falciparum* and *P. vivax* malaria?
- How self-reactive antibodies penetrate the blood-brain barrier, a high selective barrier that protects the central nervous system from invaders? Do they cross independently or do plasma cells secrete them?
- Do self-reactive antibody responses change with anti-malarial therapy?
- Is there any association between autoantibodies that persist after parasite clearance and long-term complications?
- What is the prevalence and the magnitude of autoantibody responses in different epidemiological settings?
- Can anti-self-antibody blockage prevent pathology?

These scientific breakthroughs will allow the use of autoantibodies as signatures to predict disease severity or protection, as well as provide insights toward the best vaccination strategies. Furthermore, they will open new therapeutic possibilities to treat malarial anemia.

## Concluding Remarks

Studies regarding autoantibodies and plasmodial infections have indicated that those molecules may play a dual role in malaria (Figure 1 and Table 1). However, it is not clear if self-reactive antibodies lead to pathogenesis or are just a consequence of plasmodial infection. Although different self-reactive antibodies have been identified in distinct populations and associated with clinical complications, their epitopes as well as their origin and functional role remains to be elucidated. This information will be essential to the search and identification of epitopes and other molecules that can hijacks pathogenic autoantibodies from circulation, minimizing or inhibiting their pathogenic effects in host cells. This is an interesting field of work that should be focus of future investigation using *in vitro* and *in vivo* models. Since few reports have associated autoantibodies to protection, this is an issue that should also be better investigated. Additionally, it would be of interest to determine the prevalence and the magnitude of self-reactive responses in cohorts from different epidemiological settings, an analysis that should be extended including prospective studies. The role of self-immunoglobulins isotypes and IgG subclasses is another gap that should also be addressed. A better knowledge of all these points (Box 1) may allow the use of autoantibodies as signatures to predict malaria clinical outcome. Furthermore, it may open new therapeutic possibilities to treat malaria-associated complications besides have implications for other autoimmune diseases.

**Table 1:** Autoantibodies against self-antigens and their implications in *P. falciparum* and *P. vivax* malaria.

**Self antigen**	**Possible functional activity of self-reactive antibody**	**Clinical outcome**	**References**
14-3-3 ε brain protein	Degranulation of mast cells, basophils, eosinophils and/or monocytes/macrophages	Protection against severe *Plasmodium falciparum* malaria	Duarte et al., 2012
Beta tubulin III (TBB3)	Cytoplasm microtubule disruption	Cerebral malaria associated to *P*. *falciparum*	Füchtbauer et al., 1985; Bansal et al., 2009
Dendritic tree of Purkinje cell	*In vitro* inhibition of Purkinje cells development	Cerebral malaria associated to *P*. *falciparum*	Calvet et al., 1993; Gallien et al., 2011
Erythrocyte band 3 protein	Rigidity increase and *in vitro* clearance of non-parasitized RBCs	Anemia associated to *Plasmodium vivax*	Mourão et al., 2016, 2018
	*In vitro P. falciparum* cythoadherence blockage and *in vivo* adherence of RBCs; parasite growth inhibition	Protection against *P. falciparum* malaria	Hogh et al., 1994 ; Brahimi et al., 2011
Lipids	Kidney injury through immune complex deposition	Renal failure associated to *P. falciparum* malaria	Frutakul et al., 1974; Rivera-Correa et al., 2019b
Non-erythroid alpha spectrin	Disruption of brain cells cytoskeleton; complement activation and amplification of neuronal damage	Cerebral malaria associated to *P*. *falciparum*	Guiyedi et al., 2007
Nucleic acids	Kidney injury through immune complex deposition	Renal failure associated to *P. falciparum* malaria	Frutakul et al., 1974; Rivera-Correa et al., 2019b
Phosphatidylserine	Phagocytosis (*in vitro*) and clearance of non-parasitized RBCs	Anemia associated to *P. falciparum* and *P. vivax*	Fernandez-Arias et al., 2016; Barber et al., 2019; Rivera-Correa et al., 2019a
Spectrin	Disruption of RBCs cytoskeleton; amplification of RBCs damage	Anemia associated to *P*. *vivax*	Mourão et al., 2018
Triose-phosphate isomerase	*In vitro* lysis of RBC and activation of complement	Anemia associated to *P. falciparum*	Ritter et al., 1993
Voltage-gated calcium channels (VGCC)	Complement-independent downregulation of calcium flow in Purkinge and granule cells	Cerebral malaria associated to *P*. *falciparum*	Lang et al., 2005

## Author Contributions

LM, GC-O, and EB conceptualized and wrote the manuscript.

## Conflict of Interest

The authors declare that the research was conducted in the absence of any commercial or financial relationships that could be construed as a potential conflict of interest.
